# Robo-investment aversion

**DOI:** 10.1371/journal.pone.0239277

**Published:** 2020-09-17

**Authors:** Paweł Niszczota, Dániel Kaszás

**Affiliations:** 1 Department of International Finance, Poznań University of Economics and Business, Poznań, Poland; 2 ETH Zürich, Chair of Cognitive Science, Zürich, Switzerland; Universität Heidelberg, GERMANY

## Abstract

In five experiments (*N* = 3,828), we investigate whether people prefer investment decisions to be made by human investment managers rather than by algorithms (“robos”). In all of the studies we investigate morally controversial companies, as it is plausible that a preference for humans as investment managers becomes exacerbated in areas where machines are less competent, such as morality. In Study 1, participants rated the permissibility of an algorithm to autonomously exclude morally controversial stocks from investment portfolios as lower than if a human fund manager did the same; this finding was not different if participants were informed that such exclusions might be financially disadvantageous for them. In Study 2, we show that this robo-investment aversion manifests itself both when considering investment in controversial and non-controversial industries. In Study 3, our findings show that robo-investment aversion is also present when algorithms are given the autonomy to increase investment in controversial stocks. In Studies 4 and 5, we investigate choices between actual humans and an algorithm. In Study 4 –which was incentivized–participants show no robo-investment aversion, but are significantly less likely to choose machines as investment managers for controversial stocks. In contrast, in Study 5 robo-investment aversion is present, but it is not different across controversial and non-controversial stocks. Overall, our findings show a considerable mean effect size for robo-investment aversion (*d* = –0.39 [–0.45, –0.32]). This suggests that algorithm aversion extends to the financial realm, supporting the existence of a barrier for the adoption of innovative financial technologies (FinTech).

## 1. Introduction

We are seeing a strong growth of algorithmic (systematic) investment funds [1, 2] and the robo-advisory industry [3–5]. Computer algorithms run funds that account for 35% of the US stock market, and are responsible for 60% of the trading that happens on it [6]. Investment decisions are also increasingly perceived to have moral undertones. In January 2020, the world’s biggest asset management fund declared that it would not invest in companies that receive more than a quarter of its revenues from the production of coal [7]. This move is in line with the notion that fossil fuel producers listed on stock markets might now be considered “sin stocks”: morally controversial companies whose activities cause much social harm [8, 9].

In this paper, we investigate whether people are averse to “robo” investment managers making investment decisions, and explore whether machines have a “moral mandate” that would give them legitimacy to perform investment decisions that concern controversial companies. While the study does not deal with investment mechanisms currently available to the average individual investor, progress in the robo-advisory industry suggests that more and more autonomy will be given to machines, with perhaps some of the funds being managed (almost) fully autonomously [5]. A number of automated investment services already offer “ethical” portfolios–however, at a considerable price premium due to the necessity of keeping human fund managers in the loop [10]. Thus, this is a subject worthy of investigation, somewhat similar to the case of autonomous vehicles: even though they are still in a phase that requires human-assistance (i.e., we are still many years from the full automation of road vehicles), researchers find that it is important to know what autonomous vehicles should do when facing a moral dilemma [11, 12]. Given the increasingly important role ethical and environmental considerations play in today's investment landscape [13, 14], it is worth considering if the trend towards integrating moral considerations into investment strategies and the increasing automation of investments are potentially on a collision course [15].

## 2. Hypothesis development

Recent evidence suggests that individuals show an aversion towards computer algorithms under certain conditions. When given the possibility to choose between advice provided by a human or an algorithm, people tend to display a preference for the former and thus exhibit algorithm aversion [16–19]. This algorithm aversion in the original sense can emerge in various contexts, but a common component is that people have to witness the algorithm making a mistake. In another strand of literature, Bigman and Gray [20] have demonstrated via a series of experiments that decisions with a moral component are perceived to be the domain of humans and not “machines”, showing machine-aversion in the moral domain–a tendency that was also documented by Gogoll and Uhl [21]. This effect is not predicated on the presence of previous errors, but is specific to scenarios which have a significant moral component, including the possibility of serious negative externalities (medical- and military decisions, for example).

When considering if there are further domains where decisions are often perceived to have a moral character, the world of finance and investments comes to mind. Recent evidence on how moral considerations can affect asset prices [8, 22] and experimental work on the intersection of morality and markets [23] suggest that markets and investments have an inherent moral dimension to them. The increasing demand for investments in companies representing environmentally sustainable and ethical business practices [14] shows that investors are increasingly mindful of the potential negative externalities associated with their choices. Given the parallel trend for morally sound investment practices and the automation of investment decisions [3, 4], questions emerge on the significance of algorithm aversion in investment. Our general hypothesis is that when given the decision to take investment advice from either a human or an algorithm, we expect to observe algorithm aversion. Due to the inherent moral character of investment decisions, furthermore, we hypothesize that this effect will be stronger in cases where the investment opportunities have a strong negative moral valence (“sin stocks”).

In contrast to the definition of algorithm aversion which is widely accepted in the literature [17, 18], we do not link aversion to the success or failure of the algorithms in question. Our approach is conceptually related to Bigman and Gray’s [20] and Castelo and colleagues’ [16] findings on people exhibiting a generalized aversion to machines making decisions in tasks which require subjective judgments, in our case moral judgments.

In essence, we are asking whether individual investors will be comfortable ceding control over their investments to algorithms, and if considerations of morality play a role.

## 3. Overview of experimental studies

In Studies 1–3, our main goal was to test whether people exhibit an aversion towards computer algorithms making investment decisions for them. To test this hypothesis, we implemented a between-subjects design, where participants were assigned to a condition where the fund manager was either a human or a computer algorithm. While all of these experiments were intended to test the main hypothesis, each experiment was also meant to investigate the conditions that could affect the strength of the proposed effect. In Studies 4–5, we investigate whether algorithm aversion emerges in a simulated investment scenario, where participants have to choose between real human advisors recruited via Mechanical Turk and an actual (artificial neural network) algorithm.

In Study 1, we investigated whether there is an aversion towards computer algorithms making investment decisions (which, for simplicity, we henceforth call “robo-investment aversion”), by investigating the right to exclude stocks from controversial industries from an investment portfolio. In this study, we also wanted to assess whether permissibility ratings might be different if participants are informed that excluding stocks might lead to worse performance of the portfolio. This is consistent with research from the financial literature, showing that there is a performance benefit from investing in controversial stocks (sin stocks [8]). If this was the case, then the exclusion of some controversial stocks from the portfolio might indeed hurt portfolio performance (returns).

In Study 2, we tested whether robo-investment aversion is present both when contemplating the right to exclude companies from controversial industries and non-controversial industries.

In Study 3, we further investigated how generalizable robo-investment aversion is, by simultaneously looking at the permissibility to exclude (just as in Studies 1 and 2), but also to invest more heavily in controversial companies.

In Studies 4 and 5, we pitted human advisors against an algorithm, by asking participants to choose between relatively knowledgeable humans and an artificial neural network algorithm. Crucially, we used a between-subjects design that randomly assigned participants to either controversial or non-controversial stocks, allowing us to more fully test whether an aversion towards algorithms is exacerbated in the case of controversial stocks (consistent with an attenuation in the use of algorithms for investments with moral undertones). In Study 4, we tested the preference for human versus algorithm investment advice in an incentivized experiment, where participants received a bonus if they chose the advisor that was more accurate. In Study 5, we tested whether people have a preference for knowledgeable humans over an algorithm, after finding out that they had similar overall performance in a prior task.

In all experiments, we recruited subjects through Mechanical Turk, and it is worth discussing some issues relating to this design choice. Firstly, a number of studies support the suitability of Mechanical Turk samples for experimentation (in general [24–26], and in finance-related issues [27, 28], as well). Secondly, Mechanical Turk samples seem to be more representative of the general population than student samples [29], and thus seem more suitable when representing the preferences of people that are owners of companies via pension funds or mutual funds.

Data, materials and pre-registration documents for all studies are available on OSF at https://osf.io/gdp42/?view_only=f6eeccb5e9d34f84928f58c601a7c66f.

## 4. Study 1: Exclusion of controversial stocks

In the first study, we hypothesized that people have an aversion towards computer algorithms excluding controversial companies from stock portfolios, compared to human fund managers.

### 4.1. Method

#### 4.1.1. Participants

We recruited 522 participants via Mechanical Turk, in order to obtain 90% power to detect a small-to-medium effect (*f* = .15). We excluded 56 participants who did not correctly answer an attention check question. One participant did not answer one (out of three) statements, and thus we computed the dependent variable based on the available answers. We analyzed the data of 466 participants, 261 (56%) of which were female. The mean age of participants was 39.2 years (*SD* = 11.9).

#### 4.1.2. Procedure

The study had a 2 (first between-subjects factor: portfolio choice made by human fund manager vs portfolio choice made by a computer algorithm) × 2 (second between-subjects factor: absence of penalty resulting from exclusion vs presence of penalty) design. Participants had to imagine that a fund manager or a computer algorithm (depending on which condition they are assigned to) has the autonomy to select companies to stock portfolios for moral reasons, i.e. has the autonomy to exclude (sell or not invest in) companies from a selection of controversial industries. These were presented in the first part of the study, for participants to have a reference point, using the following text:

Would you feel comfortable if your pension fund invested in companies from the following industries? Please rate on a scale of 1 (not at all) to 7 (completely).

Participants assigned to the “penalty present” condition additionally read that the exclusion of controversial stocks might lead to a slight reduction in the expected return of the portfolio. The exact instructions are presented below, with the text shown in italics being varied in human vs robo condition, and text in the square brackets being presented only to participants that were in the “penalty present” condition:

Imagine that a *fund manager/computer algorithm* had the ability to exclude (sell or not invest in) companies from some of the industries that were listed on the last page. In other words, the *fund manager/computer algorithm* would have the freedom to take into account moral issues while investing. Thus, *he/it* would have the autonomy to exclude companies with various ethical, social or environmental issues. [Also imagine that this could potentially lead to a slightly smaller expected return (e.g., slightly less money in your pension fund).]

Participants then rated–on a scale of 1 (*strongly disagree*) to 5 (*strongly agree*)–three statements adapted from Bigman and Gray [20]:

1. It is appropriate for a [fund manager/computer algorithm] to make these decisions.
2. A [fund manager/computer algorithm] should be the one to make these decisions.
3. A [fund manager/computer algorithm] should be forbidden from making these decisions.

The dependent variable–the permissibility score–was computed as the mean of the three items (after recoding the third item). To test the hypotheses, we performed a two-way ANOVA, that was meant to assess: (1) the main effect of fund manager type (first between-subjects factor) on permissibility to exclude stocks, and (2) the interaction effect between fund manager type and effect of stock exclusion (first and second between-subjects factors).

Participants in the “robo” condition were informed that “an algorithm is a sequence of computational steps that transform inputs into outputs, similar to a recipe” [30]. The full instructions are available on OSF (https://osf.io/gdp42/?view_only=f6eeccb5e9d34f84928f58c601a7c66f).

This procedure has been pre-registered (https://aspredicted.org/blind.php?x=k8wm7t) and approved by the Committee of Ethical Research conducted with participation of humans at the Poznań University of Economics and Business (Resolution 5/2019). Informed consent was obtained from all participants. Participants rated how comfortable they would feel if their pension fund invested in the controversial industries (instead of moral appropriateness; however, we use moral appropriateness in Study 2). This was the only amendment to the pre-registration (we decided to change the dependent variable, but erroneously did not incorporate this ultimate change to the pre-registration document).

### 4.2. Results

#### 4.2.1. Main analysis

In line with the main hypothesis, participants rated the permissibility to exclude stocks as higher if the fund manager was human rather than a computer algorithm (*M*_human_ = 2.95 vs *M*_robo_ = 2.64, *d* = –0.25; *F*(1, 462) = 7.83, η^2^ = .02, *p* = .005). An analysis of variance determined that there was no interaction between the two factors (*F*(1, 462) = 0.92, η^2^ = .002, *p* = .34). There were no significant differences in the permissibility ratings of people that were assigned to conditions where there was no mention of a penalty from excluding controversial stocks or a mention of such a penalty (*M*_penalty absent_ = 2.79 vs *M*_penalty present_ = 2.80, *d* = 0.004; *F*(1, 462) = 0.00, η^2^ < .001, *p* = .96).

#### 4.2.2. Additional analyses

As an addition to the pre-registered analyses, we performed a number of complementary tests relating to the general credibility of Mechanical Turk participants for carrying out tasks relating to investment in the stock market. In order to see if differences in investment knowledge affect robo-investment aversion, we collected data on participants’ subjective investment knowledge, but also constructed a six-item test to verify their objective investment knowledge, drawing on previous research [31, 32]. The former was assessed using one statement (*"My investment knowledge is good"*), whereas the latter was assessed using answers to questions such as *“Normally*, *which asset displays the highest fluctuations over time*: *savings accounts*, *bonds or stocks*?*”*. The mean subjective investment knowledge of participants (on a scale of 1 to 7) was 4.11 (*SD* = 1.60) and was not significantly different in the human and robo condition (*t*(464) = 1.50, *p* = .14). The mean score on the investment test was 4.52 (*SD* = 1.24), and was similarly not different in the human and robo condition (*t*(459) = 0.41, *p* = .68). Interestingly, the subjective and objective investment knowledge were very weakly correlated (*r* = .07, *t*(464) = 1.56, *p* = .12). While low levels of correlation between subjective and objective measures may be surprising, this is not out of line with previous research [33]. We independently investigated potential differences in robo-investment aversion for individuals with different levels of subjective (declared) and objective (actual) investment knowledge. There was no interaction between the between-individuals factor and neither subjective (*b* = –0.03, *t*(462) = –0.38, *p* = .70) nor objective (*b* = 0.07, *t*(462) = 0.83, *p* = .41) investment knowledge. Additionally, we split participants in two groups according to their level of investment experience (participants were classified as experienced if they rated themselves or scored 5 or higher; 42% (54%) of participants were in the high knowledge group based on subjective (objective) knowledge). Analyses of variance did not support the existence of an interaction between fund manager type with neither subjective (*F*(1, 462) = 0.61, η^2^ = .001, *p* = .43) nor objective *F*(1, 462) = 0.96, η^2^ = .002, *p* = .33) investment knowledge groups.

### 4.3. Discussion

The results of the study are consistent with the existence of robo-investment aversion, that is, a preference for human fund managers to make investment decisions, in this case via the exclusion of companies from portfolios. This is consequential, as exclusion serves as one form of socially responsible investment [9]. Although the existence of a penalty resulting from excluding morally controversial stocks–performing negative screening [34]–appeared to have some effect on the size of the aversion, the difference was not statistically significant. Hence, our analysis did not support the notion that people judge fund managers differently in a hypothetical setting if it is made explicit that their decision to exclude companies from portfolios might produce lower rewards (expected returns) for investors [8]. Note, however, that it is not clear what the effects of socially responsible and irresponsible investing are, that is if the former or the latter produce higher returns or there is the lack of such differences [35–37].

While some studies suggest that people might show an aversion towards machines making decisions in the moral domain [20, 21], this does not imply that there will be an absence of a bias against robo-fund managers when investing in conventional (i.e., non-sin) stocks. Therefore, in order to test whether robo-investment aversion generalizes to conventional stocks we designed Study 2, in which participants had to rate either controversial or non-controversial (conventional) companies.

## 5. Study 2: Exclusion of controversial vs non-controversial stocks

In our earlier study, we hypothesized that people have an aversion towards computer algorithms excluding morally controversial companies from portfolios (in relation to human fund managers doing the same). In Study 2, we wanted to replicate Study 1’s main finding, and also test whether the aversion towards computer algorithms is stronger in the case of controversial than non-controversial stocks. Moreover, we amended what participants were asked in the first stage of the study–instead of being asked about how *comfortable* they would feel if their pension fund invested in stocks from the shown industries, they were asked how *morally appropriate* such an investment would be.

### 5.1. Method

#### 5.1.1. Participants

We recruited 1,444 participants via Mechanical Turk, in order to obtain 90% power to detect a small effect of similar size to the more conservative estimate in our previous study (*f* = .09). We excluded 211 participants who did not correctly answer an attention check question, and additionally two participants that have answered none of the three permissibility questions used to compute the permissibility score (for five participants we computed the scores based on the available ratings). We analyzed the data of 1,231 participants, 569 (46%) of whom were female. The mean age of participants was 38.7 years (*SD* = 12.4).

#### 5.1.2. Procedure

The study had a 2 (first between-subjects factor: portfolio choice made by a human fund manager vs portfolio choice made by a computer algorithm) × 2 (second between-subjects factor: controversial stocks vs non-controversial stocks) design. Participants rated how morally appropriate it would be if their pension fund invested in companies from the list of industries that they were shown, using the following text:

Would it be morally appropriate if your pension fund invested in companies from the following industries? Please rate on a scale of 1 (not at all) to 7 (completely).

Participants were then told to imagine the investment scenario. The exact instructions are presented below, with the text shown in italics being varied in the human vs robo condition, and text in the square brackets being presented only to participants that rated controversial stocks:

Imagine that a *fund manager/computer algorithm* had the ability to exclude (sell or not invest in) companies from some of the industries that were listed on the last page. [In other words, the fund manager would have the freedom to take into account moral issues while investing. Thus, *he/it* would have the autonomy to exclude companies with various ethical, social or environmental issues.]

Afterwards, they rated the permissibility of either a human or computer algorithm to exclude companies from some of the industries. We used the same dependent variable as in Study 1. The controversial stocks we used were the seven industries that participants felt the least comfortable with in Study 1, whereas the non-controversial stocks were selected by us via a pilot study.

The procedure has been pre-registered (https://aspredicted.org/blind.php?x=cs8xe3) and approved by the Committee of Ethical Research conducted with participation of humans at the Poznań University of Economics and Business (Resolution 6/2019). Informed consent was obtained from all participants.

### 5.2. Results

#### 5.2.1. Main analysis

Prior to analyses, we performed a manipulation check, which confirmed that participants found that it would be significantly more appropriate if their pension fund invested in non-controversial stocks than in controversial stocks (*M*_controversial_ = 3.25 vs *M*_non-controversial_ = 5.55; *t*(1217) = 27.0, *p* < .001, *d* = 1.54).

Once again, consistent with our main hypothesis, participants rated the permissibility of excluding stocks as higher if the fund manager was human rather than an algorithm (*M*_human_ = 3.45 vs *M*_robo_ = 2.83; *F*(1, 1227) = 109.71, η^2^ = .08, *p* < .001, *d* = –0.58). An analysis of variance determined that there was an interaction between fund manager type and stock type (*F*(1, 1227) = 4.51, η^2^ = .004, *p* = .034), suggesting a larger difference in the permissibility ratings of human and robo fund managers for non-controversial stocks than for controversial stocks. The effect of stock type was significant, suggesting that people, in general, judge the exclusion of non-controversial stocks to be more permissible than the exclusion of controversial stocks (*M*_controversial_ = 2.96 vs *M*_non-controversial_ = 3.33; *F*(1, 1227) = 39.22, η^2^ = .03, *p* < .001, *d* = 0.34).

#### 5.2.3. Additional analyses

Similarly to what was done in Study 1, we investigated whether the degree of robo-investment aversion depended on subjective and objective investment experience. Analyses of variance provided limited support for the existence of an interaction between fund manager type and subjective investment experience group (*F*(1, 1227) = 3.73, η^2^ = .003, *p* = .054), suggesting that robo-investment aversion was stronger for participants with lower investment experience. However, there was no similar interaction with objective investment experience group (*F*(1, 1227) = 0.77, η^2^ = .001, *p* = .38).

### 5.3. Discussion

Whereas robo-investment aversion in Study 1 was equal to *d* = –0.25, in participants of Study 2 that rated controversial stocks this aversion was noticeably greater (*d* = –0.47). However, surprisingly, robo-investment aversion was also present in participants that rated non-controversial stocks (*d* = –0.74). A plausible reason for this increase in effect size is that participants in Study 2 were primed about moral norms when asked to rate how *morally appropriate* it would be if their pension fund excluded investments in companies from the seven industries presented to them. In contrast, in Study 1 they had to rate how *comfortable* they would feel, which might not necessarily prime moral norms. Also, in Study 2 we used a subset of controversial industries with which participants felt the least comfortable with and had a high potential to violate social norms, which further facilitated the moral aspect of allocating funds in companies listed on stock markets. While there are relatively clear-cut moral concerns in the case of controversial industries, this is less so the case when it comes to traditionally non-controversial companies. In order to judge the moral appropriateness of investing in such companies, one might need a higher level of moral competence–one that machines are not expected to possess. Finally, participants were not informed that the exclusion of stocks might lead to worse returns (as were half the participants in Study 1).

## 6. Study 3: Exclusion vs heavier investment in controversial stocks

In both previous studies, we hypothesized that people have an aversion towards computer algorithms excluding companies from stock portfolios compared to human fund managers. In our third study, we wanted to test whether apart from an aversion to exclude controversial stocks (as earlier), there is also an aversion towards computer algorithms to invest more heavily in controversial stocks. Therefore, we used a mixed design, where the type of fund manager (human vs robo) was the between-subjects factor (similarly to Study 1 and 2) and decision type (exclusion or heavier investment) was the within-subjects factor. While one might consider exclusion and inclusion opposite sides of the same coin, recent research on the psychological differences between buying and selling decisions [38, 39] hints at the potential existence of asymmetries between how investors approach positive- and negative screening. This might especially be the case when it comes to morally controversial decisions [40].

### 6.1. Method

#### 6.1.1. Participants

We recruited 722 participants via Mechanical Turk. Although this was a significantly lower sample size than the one used in the previous study, one of the factors was within-subjects, which allowed us to retain 90% power from the former studies much more efficiently (with less participants). This change was also intended to test of the robustness of our effect to changes in the design, similar to other studies on machines [20]. We excluded 39 participants who did not correctly answer an attention check question. We analyzed the data of 683 participants, of which 387 (57%) were female. The mean age of participants was 39.3 years (*SD* = 12.0).

#### 6.1.2. Procedure

Participants assessed the moral appropriateness of their mutual fund holding 14 controversial industries (the same industries that we used in Study 1). We changed the investment fund type to account for the eventuality that this might be more believable to some participants (and, at the same time, to test the robustness of our earlier findings). After rating the industries, participants assessed the permissibility of their mutual fund excluding and investing more heavily in companies from some of the controversial industries (the order of presentation was counter-balanced). The design and wording of this study was similar to Studies 1 and 2, but as part of the newly added within-factor, participants were asked not just about the permissibility of excluding certain stocks, but also about investing more heavily in certain stocks.

We used the same dependent variable as in Studies 1 and 2. We performed a mixed ANOVA, that was meant to assess: (1) the main effect of fund manager type (between-subjects factor) on permissibility, (2) the main effect of decision type (the within-subjects factor) on permissibility, and (3) the interaction effect between fund manager type and decision type.

This procedure has been pre-registered (https://aspredicted.org/blind.php?x=d5fg7a) and approved by the Committee of Ethical Research conducted with participation of humans at the Poznań University of Economics and Business (Resolution 6/2019). Informed consent was obtained from all participants.

### 6.2. Results

#### 6.2.1. Main analysis

**Fig 1:**
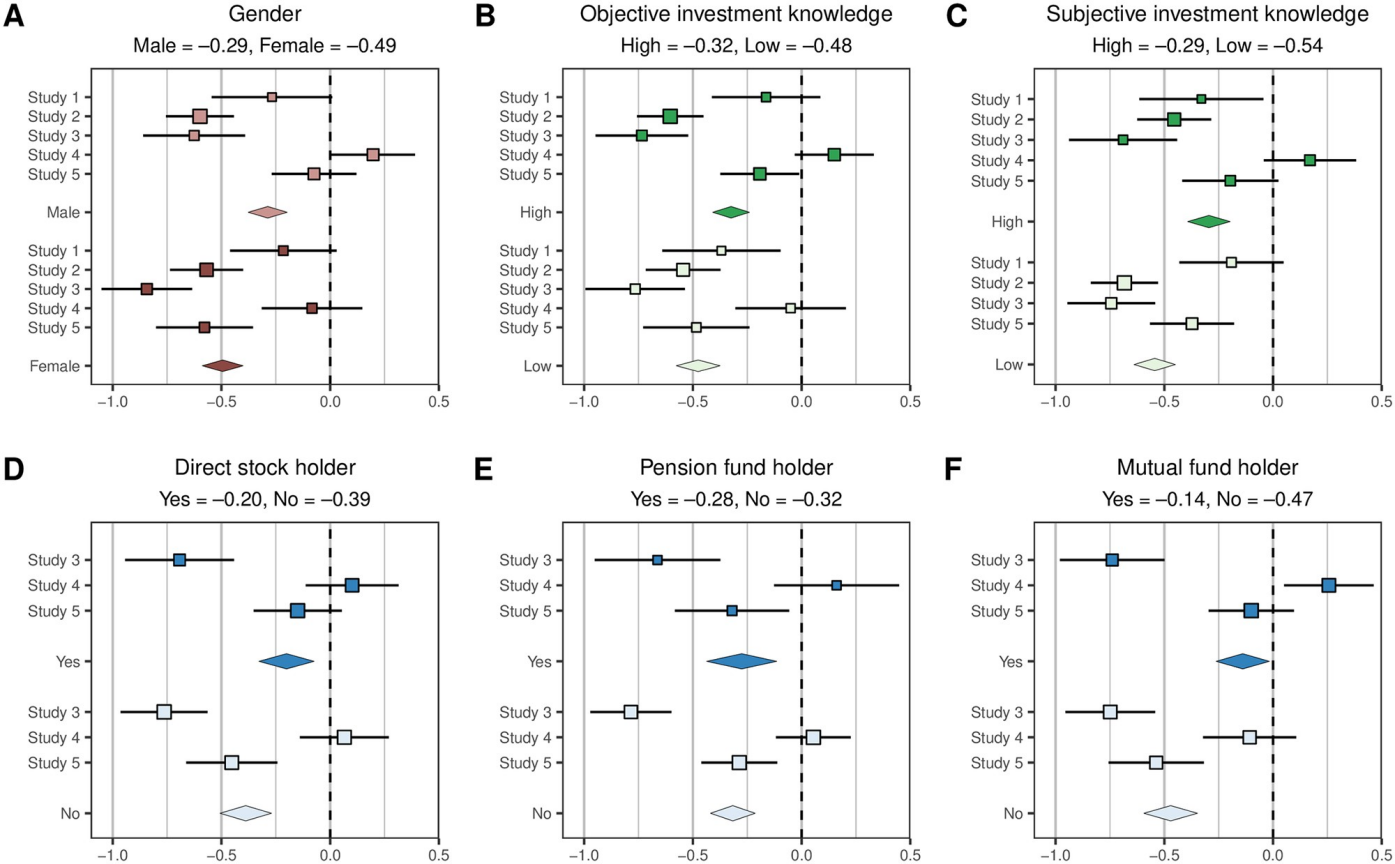
Robo-investment aversion across subsamples. Effects sizes are presented as Cohen's *d*s. Mean effect sizes are fixed effects, computed using the *R* package *metaviz* [58,59]. Error bars correspond to 95% confidence intervals. All mean subgroup effects are significant at the 5% level.

An analysis of variance showed that, in general, participants rated computer algorithms to be less permitted to make investment decisions than humans (*M*_human_ = 3.48 vs *M*_robo_ = 2.58; *F*(1, 681) = 130.38, η^2^ = .14, *p* < .001, *d* = –0.81). Robo-investment aversion (see Fig 1C) was present both when fund managers had the autonomy to exclude controversial stocks (*M*_human_ = 3.46 vs *M*_robo_ = 2.64, *t*(671) = 9.72, *p* < .001, *d* = –0.75), and when they had the autonomy to invest more heavily in such stocks (*M*_human_ = 3.50 vs *M*_robo_ = 2.53, *t*(665) = 11.50, *p* < .001, *d* = –0.88). The type of operation (exclusion vs heavier investment) did not have a statistically significant effect on permissibility ratings (*F*(1, 682) = 0.97, *p* = .33). However, the interaction between the between-subjects factor (human vs robo fund manager) and within-subjects factor (autonomy to exclude stocks vs autonomy to invest more heavily in stocks) was statistically significant (*F*(1, 681) = 4.77, η^2^ = .001, *p* = .029), consistent with robo-investment aversion being stronger when fund managers had the autonomy to invest more heavily in controversial stocks than when they had the autonomy to exclude them.

#### 6.2.2. Additional analyses

In Study 3 we collected data on whether participants held stocks directly and via pension- or mutual funds, in order to see if robo-investment aversion manifests itself in experienced participants. Robo-investment aversion was present both in participants that held stocks directly (*d*_stocks_ = –0.73) and those that didn’t (*d*_no stocks_ = –0.85). Similarly, robo-investment aversion appeared in participants with or without mutual- (*d*_mutual funds_ = –0.77 vs *d*_no mutual funds_ = –0.83) or pension- (*d*_pension funds_ = –0.73 vs *d*_no pension funds_ = –0.86) fund holdings. In none of the cases was there an interaction between fund manager type and the holding of stocks or funds (*p*s > .43). Separate analyses of variance showed that there was no interaction between fund manager type and subjective (*F*(1, 679) = 0.86, η^2^ = .001, *p* = .35) or objective investment knowledge group (*F*(1, 679) = 0.05, η^2^ < .001, *p* = .82).

### 6.3. Discussion

In Study 3 we investigated whether robo-investment aversion generalizes to investment decisions, in which fund managers decide to allocate *more* in companies from controversial industries. Consistent with Studies 1 and 2, participants exhibited an aversion towards algorithms making decisions about increased investments in controversial stocks. In fact, the aversion was higher when the fund managers had heavier-investment autonomy instead of exclusion autonomy. Overall, this supports the robustness of the investigated effect, suggesting that algorithmic investment funds might be considered a less legitimate way of investing in companies from controversial industries. This suggests that computerized funds might *also* have lower legitimacy to perform socially responsible investing (e.g., excluding or investing more heavily in “saint” stocks; [22]). This prediction was not tested by us but might be of interest to other researchers.

We summarize permissibility ratings in Studies 1–3 in Table 1.

**Table 1:** Permissibility to make investment decisions.

Study	Second factor	Human	Robo	*d*
Study 1	Penalty absent	3.01 (1.22)	2.59 (1.16)	–0.35 [–0.62, –0.08]
	Penalty present	2.90 (1.24)	2.69 (1.19)	–0.17 [–0.42, 0.08]
	**Overall**	**2.95 (1.23)**	**2.64 (1.17)**	**–0.25 [–0.44, –0.07]**
Study 2	Controversial	3.22 (1.07)	2.71 (1.11)	–0.47 [–0.62, –0.31]
	Non-controversial	3.73 (1.01)	2.96 (1.05)	–0.74 [–0.91, –0.57]
	**Overall**	**3.45 (1.07)**	**2.83 (1.09)**	**–0.58 [–0.69, –0.47]**
Study 3	Exclusion	3.46 (1.09)	2.64 (1.14)	–0.75 [–0.90, –0.59]
	Heavier investment	3.50 (1.05)	2.53 (1.14)	–0.88 [–1.04, –0.72]
	**Overall**	**3.48 (1.07)**	**2.58 (1.14)**	**–0.81 [–0.92, –0.70]**

All studies reported in this table have a 2 × 2 design, with human vs robo investment manager being the first factor, and the second factor reported in the second column. *SD*s are reported in parentheses. *d* denotes Cohen’s *d*; 95% confidence intervals are reported in the square brackets.

Studies 1–3 relied solely on hypothetical choices. In order to test actual decisions–as it is often performed in the literature on algorithm aversion [17, 41]–in Studies 4 and 5 participants had to decide whether they would prefer humans or an algorithm as advisors in an investment task that mimicked investment reality.

## 7. Study 4: Incentivized choice between human and robo investment managers

In Study 4 we pitted humans against algorithms as investment advisors. In this study we tested in an incentive-compatible manner whether people exhibit algorithm aversion when asked to decide whether they would want actual humans or an actual algorithm to serve as advisors in a simplified investment task. Additionally, the design of the study allows us to once again directly compare whether participants’ choices differ for controversial and non-controversial stocks.

Study 4 is conceptually similar to studies on algorithm aversion by Dietvorst and colleagues [17]. The design has also been guided by Önkal and colleagues [42].

### 7.1. Participants

We recruited 838 participants via Mechanical Turk, allowing us to obtain 80% power detect a 10% percentage point difference in choice (between 55 and 65%, set arbitrarily). We excluded 133 participants who did not correctly answer an attention check question. We analyzed the data of 705 participants, of which 288 (41%) were female. The mean age of participants was 39.4 years (*SD* = 12.3).

### 7.2. Procedure

Participants saw a series of historical price paths [43, 44] and were asked to decide whether they would prefer their investments in these stocks to be managed by a group of humans with investment knowledge, or by an algorithm (using the *nnetar* function from the *forecast* package in *R* [45], which trains a neural network on the historical price paths to forecast future prices). The knowledgeable humans were the 11 most effective performers in a pilot study (*N* = 101), carried out earlier on Mechanical Turk: we assessed their investment knowledge using a very brief test (with a maximum score of 3; see Experimental Materials on *OSF* for details), and then told them to predict the price of the stocks two months after the shown 1-year period (252 trading days). We used actual stock prices, but not from the most recent year, to make it harder to identify the price paths from memory. Participants were told that if they chose the advisor that was more accurate (on average) in predicting the stock prices, they would get a bonus, doubling the compensation for participating in the task.

The experiment had a 2-cell design, where participants were shown the price paths of controversial or non-controversial stocks.

This procedure has been pre-registered (https://aspredicted.org/blind.php?x=ak6th4).

### 7.3. Results

A similar proportion of participants chose human (48%) over algorithm (52%) advice, and thus–in contrast to the earlier studies–there was no evidence of an aversion towards algorithms in the entire sample (χ^2^(1) = 1.19, *p* = .27; *d* = 0.08). However, the choice of advisor differed in controversial stocks (where 47.8% chose the algorithm; *d* = –0.09) and non-controversial stocks (where 56.5% chose the algorithm; *d* = 0.26), showing a greater reliance on human advice for controversial stocks (χ^2^(1) = 5.37, *p* = .021; *d* = 0.18).

#### 7.3.1. Additional analyses

The effect was of similar magnitude for participants that held stocks directly (*d*_stocks_ = 0.10) and those that didn’t (*d*_no stocks_ = 0.07), but the preference for machines was not statistically significant at conventional levels. The same pattern appeared in participants with or without pension fund holdings (*d*_pension funds_ = 0.16 vs *d*_no pension funds_ = 0.05). However, algorithm (machine) appreciation was significant in participants that had mutual fund holdings, in contrast to participants that did not have such holdings (*d*_mutual funds_ = 0.26 vs *d*_no mutual funds_ = –0.11).

### 7.4. Discussion

In contrast to the results of the earlier studies, in Study 4 we found no general aversion towards algorithms, but a difference in the utilization of human advice depending on whether advice concerned morally controversial investments or not. This is consistent with the notion that people might be more reluctant to use robo-investment funds for morally controversial investments. A potential explanation for why we could not produce general aversion towards algorithms in Study 4 might be due to differences in the framing and design of the experimental task. While Studies 1–3 concern the morality of investment decisions (with a potential impact on returns), Study 4 concerns performance in a forecasting task between human and algorithmic agents.

A plausible reason for the lack of algorithm aversion is that perhaps a considerable number of people consider algorithms to have superior performance over (knowledgeable) humans, at least in this particular task. While we do not believe that such beliefs are responsible for driving the observed effects, we nevertheless decided to conduct an additional study to account for this possibility. To address this potential alternative explanation, in Study 5 we equalize the expected outcomes of humans and the artificial neural network algorithm, allowing us to investigate whether people will prefer humans over algorithms after making it explicit that there are no differences–on average–between them.

## 8. Study 5: Choice between human and robo investment managers after outcomes are equalized

In Study 5 we utilized the same design that we used in Study 4, with a key difference: participants were truthfully informed that the (knowledgeable) humans–that scored a maximum (3 out of 3) score on the investment knowledge test in the same pilot study that we describe in Study 4 –performed similarly to the (artificial neural network) algorithm. However, participants were also informed that humans and the algorithm might differ in their accuracy to predict future prices of certain industries or particular stocks. This was intended to set the conditions for the final test of the hypothesis that the reliance on humans as investment managers will be stronger for controversial than non-controversial stocks, assuming that the industry itself is a strong enough cue to guide participants’ moral compasses.

### 8.1. Participants

We recruited 882 participants via Mechanical Turk. Similarly to Study 4, we wanted to detect a 10 percentage point difference, but collected more data to account for a higher (15%) expected exclusion rate of inattentive participants. We excluded 139 participants who did not correctly answer an attention check question. We analyzed the data of 743 participants, of which 335 (45%) were female. The mean age of participants was 40.3 years (*SD* = 12.5).

### 8.2. Procedure

Participants were informed that the performance of humans and computers is similar (for all stocks), but that humans and computers can have a different level of accuracy for some stocks (or industries).

This experiment had a 2-cell design: participants were either allocated to the controversial stocks condition or the non-controversial stocks condition, in which they saw the historical price paths of five (sin or conventional) stocks. We used the past performance of stocks that represented each of these industries. The stocks were selected so that the past performance of the portfolios of sin and conventional stocks was similar.

This procedure has been pre-registered (https://aspredicted.org/blind.php?x=nt9n9j).

### 8.3. Results

Participants showed a preference for human advice, choosing them in 57.3% of cases, which was significantly different from 50% (χ^2^(1) = 16.0, *p* < .001). This is consistent with robo-investment aversion (*d* = –0.30).

Participants showed an aversion of similar magnitude for controversial stocks (human advice was preferred by 57.0% of participants) and non-controversial stocks (human advice was preferred by 57.6% of participants; χ^2^(1) = 0.03, *p =* .87).

#### 8.3.1. Additional analyses

*Pooled analysis*. Considering the very similar structure of Studies 4 and 5, we also performed an analysis on the pooled dataset. Overall, 47.0% of participants in these studies preferred the algorithm over humans, which is consistent with robo-investment aversion (χ^2^(1) = 4.42, *p* = .036). This aversion was not different across controversial and non-controversial stocks (χ^2^(1) = 1.95, *p* = .16).

*Effect of differences in investment experience*. In Study 5, robo-investment aversion was present both in participants that did not hold stocks or mutual funds (*d*_no stocks_ = –0.45; d_no mutual funds_ = –0.54), but not in those that did (*d*_stocks_ = –0.15; *d*_mutual funds_ = –0.10). However, robo-investment aversion was of similar magnitude for both participants that did or did not hold pension funds (*d*_pension funds_ = –0.29 vs *d*_no pension funds_ = –0.35).

*Text analysis*. In addition to testing our hypotheses, we wanted to further investigate the beliefs motivating our participants to choose humans or an algorithm. As part of a pre-registered analysis, we gave our participants the option to explain their decision, and incentivized them by offering a lottery bonus of $20. Anyone submitting an original and intelligible contribution participated in the lottery, regardless of their stated reasons for selecting one option or the other. Six-hundred and twenty-two participants opted to participate, yielding a combined output of 23,721 words. Excluding English stop words [46] and words which were contained in the original question formulation (*“Please tell us why you chose the sophisticated (neural network) algorithm / Please tell us why you chose the knowledgeable humans”*) resulted in 10,741 words.

We find that individuals choosing the algorithm use the terms “bias” and “emotions” eleven- and three times more often than the human-choice group, respectively. This indicates that individuals who chose the algorithm might have done so in part due to a belief that algorithms are less influenced by factors extraneous to the investment decision. In a subsequent bigram analysis for this subgroup, “human” is the word most often associated with “emotion” or “emotions” (*N* = 19), supporting this interpretation. Similarly, the word most often associated with “bias” or “biases” is “human” (*N* = 8). On the other hand, participants who preferred to follow human investment advice made four times as many references to “industries”, presumably as a reference to the type of industries presented in the respective conditions. Further terms more associated with choosing humans were “knowledge”, “experience” and “factor”–all most often preceded by the term “human” in a bigram analysis. What this analysis reveals is that even though participants were explicitly told to expect similar prediction performance from both human- and algorithmic decision-makers, they still express diverse beliefs when it comes to the process leading to these similar outcomes.

### 8.4. Discussion

In contrast to Study 4, in Study 5 the results point to the existence of robo-investment aversion, but an aversion that is not different across controversial and non-controversial stocks. A pooled analysis for Study 4 and 5 supports the existence of robo-investment aversion, but does not suggest the existence of differences in this aversion between controversial and non-controversial stocks. Note, however, that Studies 1–3 and Studies 4–5 are different in their nature. In the former, participants judged the permissibility of either a human or robo to “call the shots” while excluding (or investing more heavily) in companies. In contrast, in Studies 4–5, participants are asked whether they would prefer (knowledgeable) humans or a (neural network) algorithm for predictions of future stock prices. If people show a preference for a human over a robo investment fund manager (a long-term, complex task, requiring a series of judgments of various nature), it is not necessarily the case that this preference will manifest itself in a much simple task of predicting stock prices based on past stock prices alone.

## 9. General discussion

The dawn of algorithm-generated (“robo”) advisors warrants us to ask how people judge the perspective of machines making investments for them (“robo-investments”), some of them in morally controversial stocks [3, 8]. Previous research usually documents an aversion towards algorithm use [17, 18], yet under some circumstances the opposite is true [41], This suggests that algorithm aversion should not be universally expected. In our case, we test whether it extends to investments, focusing mainly on investments in morally controversial stocks. In five experiments (*N* = 3,828)–summarized in Table 2 –we document a considerable robo-investment aversion (*d* = –0.39 [–0.45, –0.32] in internal meta-analysis [47]).

**Table 2:** Robo-investment aversion across studies.

Study	*N*	Design	Dependent variable	Effect size < 0 indicates robo-investment aversion		
				Controversial stocks	Non-controversial stocks	Pooled
1	466	2 (between-subjects: human vs robo) × 2 (between-subjects: penalty vs no penalty)	Permissibility to exclude (Studies 1–3) or invest more heavily (Study 3) in stocks *1 = strongly disagree 5 = strongly agree*	–0.25 [–0.44, –0.07]	–	–0.25 [–0.44, –0.07]
2	1,231	2 (between-subjects: human vs robo) × 2 (between-subjects: controversial vs non-controversial)		–0.47 [–0.62, –0.31]	–0.74 [–0.91, –0.57]	–0.58 [–0.69, –0.47]
3	683	2 (between-subjects: human vs robo) × 2 (within-subjects: exclusion vs inclusion)		–0.81 [–0.92, –0.70]	–	–0.81 [–0.92, –0.70]
4	705	2-cell (between-subjects: controversial vs non-controversial)	Choice of investment manager *= 1 robo* *= 0 human*	–0.09 [–0.30, 0.12]	0.26 [0.05, 0.47]	0.08 [–0.07, 0.23]
5	743	2-cell (between-subjects: controversial vs non-controversial)		–0.31 [–0.51, –0.11]	–0.28 [–0.49, –0.07]	–0.30 [–0.44, –0.15]
**1–5**	**3,828**					**Mean effect** fixed effects model **–0.39 [–0.45, –0.32]**
						**Mean effect** random effects model **–0.36 [–0.64, –0.08]**

Mean effect sizes are Cohen’s *d*s, with 95% confidence intervals reported in square brackets. Computed using the *R* package *metaviz*, based on the *metafor* package [58, 59]. Random effects are computed using the *REML* method.

Comparisons of the strength of the aversion for controversial and non-controversial stocks–defined based on the industry in which they operate–suggest that it might even be stronger for the latter (Study 2), or that the two are not different (Study 5). However, this does not necessarily rule out the role moral concerns play when choosing between a human and robo investment manager or advisor. Note that this finding might simply reflect the fact, that it is more difficult to make judgments in the case of non-controversial than controversial industries. Robos can be easily programmed to exclude companies from some (or all) sin industries if this is the wish of the investor, who will surely not question the robos’ ability to follow such a simple rule. Put differently, some participants might perceive the industry in which the company operates as a sufficient “moral cue”, which makes further investigation of a controversial company by a competent moral examiner (a human; [20]) redundant. For companies from non-controversial industries, however, choosing the “sinners” is a more difficult task. An investor that cares about moral issues will want the investment manager (advisor) to have the additional ability to identify the “bad apples” in industries that are not known to be controversial, at least relative to other industries. For example, it is probably fair to say that the car industry produces–relative to the tobacco industry–less harmful (more morally acceptable) goods. In such a case, people might prefer their money to be managed by a human fund manager, who will probably be perceived to be more competent to handle less conventional cases. A counter-argument of increasing prominence is that although moral issues seem to be more “fuzzy” (more difficult to quantify) in general, it is now easier for algorithms to monitor the “moral risk” of potential investments, taking into account scores for sustainability [48] or corporate governance, and synthesizing it with the emotional valence of news or social media content [49–51]. A number of startups have recently started offering such services [10, 15]. Yet, the availability of “hard” data on responsible business practices in itself is insufficient to solve the problem. There are multiple agencies that rate what environmental, social and governance policies companies have in place (or lack). The ratings provided by competing agencies are diverging [52]. Choosing the “right” agency is a task where, once again, people will probably gravitate towards human decision-makers (and not robos).

Understanding the personal and demographic variables associated with robo-investment aversion is a further step towards understanding the role such differences might play in technology adoption in the domain of finance. In a secondary analysis we find that robo-investment aversion is present among males and females (Fig 1, Panel A), in people with high or low investment knowledge (both objectively- and subjectively-measured; Panels B-C), and regardless of whether a person holds stocks in their portfolios (either directly or via pension or mutual funds; Panels D-F). Interestingly, the (exploratory) analysis revealed that robo-investment aversion was stronger for female participants, consistent with a multitude of studies documenting gender differences in investment behavior (e.g., [53–55]). The aversion was also stronger in participants with lower investment knowledge, and in participants that do not hold stocks directly or via mutual funds (*p*s < .05). This is perhaps unfortunate, as one of the benefits of robo-advice is that is lowers the barriers-of-entry into financial investments, making it easier to enjoy the benefits of stock market participation.

A limitation of our paper is that we have incentivized the choice of investment advisor in only one of our studies (Study 4; in Study 5 the incentives were used only to promote thoughtful responses in an exploratory study of the motivations behind participants’ choices), and it happens to be the only study where there was no bias against algorithms. However, we believe this is most likely due to the belief of some participants that algorithms produce superior outcomes, in accordance with reality [17]. Bias against algorithms re-emerges once participants are informed that humans and algorithms (in our case: an artificial neural network) produce similar outcomes, (presumably) taking away the algorithms’ perceived edge [16].

Overall, our study suggests the existence of a barrier for the adoption of innovative financial technologies (FinTech; e.g., [56]; however, see [21] for an alternative account). While investors today enjoy an unprecedented ease of access to financial products and services, they might still be reluctant to use them. Our findings suggest the future proliferation of hybrid systems [4], considering mounting pressures to reduce funds’ costs of operation. The need for supervision by humans can be seen as a pitfall for the robo-advisory services [3]. A potential problem that might arise in the future would be the overreporting of human fund manager presence, to lower costs. However, finding out that investment funds have been managed by machines might undermine trust, given the evidence of the emergence of a bias against robots in certain scenarios [57].

To summarize, we find that people display an aversion to investing with algorithms. Our findings can be interpreted as follows: people perceive algorithms as being less effective than humans at tasks which require making subjective judgments [16]. Future research could investigate the drivers of algorithm aversion in the domain of investments, including a more systematic exploration of the role of moral considerations and situational factors, and a further examination of the role of individual differences.
